# Whole-genome sequencing confirms *Shigella flexneri* infection in a child with shock and suspected multisystem inflammatory syndrome in children: A case report

**DOI:** 10.1016/j.idcr.2025.e02274

**Published:** 2025-05-28

**Authors:** Sheridan J.C. Baker, Laura K. Erdman, Donald Brody Duncan, Tania Cellucci, Candy Rutherford, Marek Smieja, Cheryl Main, Jeffrey M. Pernica, Andrew G. McArthur

**Affiliations:** a Department of Biochemistry & Biochemical Sciences, McMaster University, 1280 Main Street West, Hamilton, Ontario L8S 4K1, Canada; b Michael G. DeGroote Institute for Infectious Disease Research, McMaster University, 1280 Main Street West, Hamilton, Ontario L8S 4K1, Canada; c Division of Pediatric Infectious Disease, McMaster Children’s Hospital, 1280 Main Street West, Hamilton, Ontario L8S 4K1, Canada; d Department of Pathology and Molecular Medicine, McMaster University, 1280 Main Street West, Hamilton, Ontario L8S 4K1, Canada; e Division of Pediatric Rheumatology, McMaster Children’s Hospital, 1280 Main Street West, Hamilton, Ontario L8S 4K1, Canada; f Hamilton Regional Laboratory Medicine Program, Hamilton Health Sciences, 50 Charlton Ave E, Hamilton, Ontario L8N 4A6, Canada

**Keywords:** *Shigella*, Multisystem inflammatory syndrome in children, Shock, Whole-genome sequencing, Case report

## Abstract

**Background:**

Differentiating severe systemic inflammatory syndromes from sepsis can be challenging. The diagnostic process may be further complicated by concurrent infection and hyperinflammation, with important management implications. We report a child with suspected multisystem inflammatory syndrome in children, who was unexpectedly diagnosed with *Shigella* gastroenteritis.

**Case presentation:**

A previously healthy 6-year-old boy acutely presented with fever, vomiting, diarrhea, fluid-refractory shock, cardiac dysfunction, biochemical inflammation, and coagulopathy. He fulfilled diagnostic criteria for multisystem inflammatory syndrome in children, including SARS-CoV-2 exposure 8 weeks prior. He received both antibiotics and pulsed intravenous methylprednisolone, with rapid improvement. Stool molecular testing using a lab-developed multiplex qPCR assay revealed *Shigella flexneri* infection, confirmed by culture, MALDI-TOF mass spectrometry, and whole-genome sequencing. Serologic testing confirmed prior infection by the SARS-CoV-2 virus. The child fully recovered. Immunological investigations were normal. The case was investigated by the Public Health Department, but the source of *Shigella* infection was not identified.

**Conclusions:**

This case underscores the importance of systematic microbiological workup in suspected systemic inflammatory syndromes, to identify infections that are treatable and of public health relevance. Given the rarity of *Shigella* septic shock in immunocompetent individuals, this case raises the possibility that recent SARS-CoV-2 infection led to immune dysregulation and exaggerated inflammatory responses to *Shigella*. It also demonstrates the utility of molecular testing for rapid diagnosis and confirmation of gastrointestinal infection.

## Highlights

• *Shigella* septic shock can mimic Multisystem Inflammatory Syndrome in Children.
• Molecular stool analysis was critical for rapid and accurate diagnosis of infection.
• Preceding SARS-CoV-2 infection may have contributed to severity of shigellosis.

## Introduction

Severe infectious and inflammatory syndromes can both present with acute onset of fever, multi-system inflammation, and organ dysfunction. These include septic shock, toxic shock syndromes, Kawasaki disease (KD), hemophagocytic lymphohistiocytosis, and multisystem inflammatory syndrome in children (MIS-C). Rapid and accurate diagnosis is critical to prevent progression to severe and fatal disease. Differentiating between these entities is challenging, given significant overlap in clinical and biochemical parameters [1], [2], Vogel et al., 2021. To further complicate the diagnostic process, hyperinflammation can be triggered by a recent or concurrent infection.

MIS-C is a rare hyperinflammatory condition that follows natural SARS-CoV-2 infection. Common clinical features include fever, rash, mucosal changes, gastrointestinal symptoms, and cardiovascular instability. The spectrum of MIS-C ranges from a mild febrile illness to life-threatening hypotension and multi-organ dysfunction. Negative microbiological testing and rapid clinical response to high-dose steroids help distinguish MIS-C from sepsis, but initial presentation can be similar. A proportion of suspected MIS-C cases are ultimately diagnosed with treatable bacterial infections, such as bacteremia, abscess, lymphadenitis, urinary tract infection, and bacterial gastroenteritis, including *Salmonella* and *Campylobacter* infections [3], [4], [5], [6]. Diagnosing a bacterial etiology in such cases is important for ensuring appropriate antibiotic treatment, and identifying pathogens of public health relevance.

*Shigella* is a genus of gram-negative bacillus transmitted via the fecal-oral route, through close contact with infected individuals or contaminated food or water. Healthy individuals may have mild diarrhea, or progress to bloody or mucoid stools if the colonic epithelium is invaded. Severe disease can occur in high-risk groups such as children under 5 years old, the elderly, and malnourished or immunocompromised individuals. Severe manifestations of shigellosis include profound dehydration, bacteremia, bowel perforation, and neurological complications [8]. *Shigella* is a major cause of child morbidity and mortality in low- and middle-income countries, but is relatively rare in industrialized countries [9]. In Canada, the annual incidence of shigellosis is approximately 2/100,000 [10]. Many *Shigella* cases are travel-acquired, though local transmission also occurs [11].

We report a child presenting in shock shortly after the SARS-CoV-2 Omicron wave, with strong suspicion for MIS-C given typical clinical and biochemical features and recent contact with a COVID-19 case. Unexpectedly, molecular stool testing revealed *Shigella flexneri* infection.

## Case

A previously healthy, well-grown 6-year-old boy presented to the Emergency Department in March 2022. The previous day, he had developed tactile fever, headache, vomiting, and abdominal pain. On the day of presentation, he had profuse non-bloody diarrhea and lethargy. There was no rash, conjunctivitis, or mucosal changes.

On assessment, he was febrile (39.2 °C), hypotensive, and fatigued, with mild abdominal tenderness. He received normal saline boluses totaling 60 mL/kg followed by an epinephrine infusion to maintain blood pressure. Ceftriaxone and vancomycin were initiated following collection of blood, urine, and stool cultures. Laboratory investigations revealed normal hemoglobin and platelets, leukopenia (2.1 × 10^9^ cells/L), neutropenia (0.4 × 10^9^ cells/L), lymphopenia (0.7 × 10^9^ cells/L), and hypoalbuminemia (18 g/L). He had elevated C-reactive protein (184 mg/L), creatinine (89 umol/L), INR (3.3), troponin (49 ng/L), and NT-proBNP (21,681 ng/L).

Over the next hours, his epinephrine infusion was increased due to ongoing shock, and norepinephrine infusion was added. Echocardiogram demonstrated normal coronary arteries and mildly reduced systolic ejection fraction of 50 %.

The Rheumatology and Infectious Disease services were consulted, and further history was obtained. Approximately 2 months prior, during the SARS-CoV-2 Omicron wave, the child had experienced mild upper respiratory tract symptoms. The family reported a very faint band on a rapid SARS-CoV-2 antigen test at the time. Other family members had experienced similar symptoms, and a cousin (with whom the child had regular contact) had a positive rapid SARS-CoV-2 test. The child had never been vaccinated against COVID-19. He immigrated from Nigeria in 2019, and had no subsequent travel outside of Canada. The week prior, he attended a community event at an airport hotel. There were no known sick contacts, nor consumption of undercooked meat.

As the child met diagnostic criteria for MIS-C [3], he was initiated on pulsed intravenous methylprednisolone (30 mg/kg), intravenous immunoglobulin (2 g/kg single dose), and thromboprophylaxis with low molecular weight heparin. Ceftriaxone was broadened to meropenem. Within 2 h of methylprednisolone initiation, and 17 h after antibiotic initiation, his blood pressure stabilized, and inotropic support was rapidly weaned.

Later that day, stool multiplex polymerase chain reaction (PCR) testing yielded a positive result for the *ipaH* target shared by *Shigella spp.* and enteroinvasive *Escherichia coli* (EIEC), and negative results for Shiga-toxin genes *stx1* and *stx2, E. coli* O157:H7, *Salmonella spp.*, *Yersinia enterocolitica,* and *Campylobacter coli/jejuni,* adenovirus, rotavirus, and norovirus. Stool was tested using a lab-developed q-PCR Taqman assay performed on the BD MAX instrument (Becton Dickinson, Mississauga, ON, Canada) using Luna® Universal Probe qPCR Master Mix (New England Biolabs, Whitby, ON, Canada) for bacterial targets, and qRT-PCR for viral targets. Primers and probes were manufactured by Biosearch Technologies, Middlesex, UK. Blood and urine cultures were negative. Nasopharyngeal swab for respiratory viruses was negative using a lab-developed multiplex qPCR assay on the BD MAX instrument, which tests for SARS-CoV-2, influenza A virus, influenza B virus, respiratory syncytial virus, human metapneumovirus, parainfluenza virus type 3, adenovirus, and rhinovirus/enterovirus.

Antibiotics were narrowed to ceftriaxone for total duration 3 days for possible severe shigellosis. The child received pulse steroids for 3 days, then transitioned to oral prednisone. He improved clinically and biochemically, and ventricular function normalized. Serum IgG for SARS-CoV-2 anti-nucleocapsid and anti-spike proteins were positive, suggestive of previous infection.

Stool was cultured on Hektoen media and grew colonies with a preliminary identification of *Shigella* by Vitek II GNI card (bioMérieux Canada, Saint-Laurent, QC, Canada). The isolate was sent to the provincial public health lab for final identification. In the interim, we initiated whole-genome sequencing of the isolate for further characterization given the shared *Shigella*/EIEC target, rarity of septic shock secondary to gastroenteritis, and MIS-C as an alternative diagnosis. Briefly, genomic DNA was extracted using a Promega (Madison, WI, USA) Wizard Genomic DNA purification kit according to manufacturer’s specifications. Genomic DNA was then fragmented in Ultra II first strand buffer (New England Biolabs, Whitby, ON, Canada) and Ultra II first strand enzyme for 3 min and 45 s at 37°C before 30 min at 65°C. NEB adaptor was then ligated using Ultra II ligation master mix and enhancer and incubating for 15 min at 20°C. Ligated DNA was treated with Uracil-Specific Excision Reagent (NEB) for 15 min at 37°C before ProNex bead (Promega) purification. NEB i7 and i5 barcodes were then ligated to the DNA before a final ProNex bead purification. The sample was sequenced using 2 × 150 bp paired-end sequencing on an Illumina NextSeq (San Diego, CA, USA). After sequencing, FASTQ files were analyzed via FastQC (bioinformatics.babraham.ac.uk/projects/fastqc/). Barcode and adaptor sequences were removed using Trimmomatic [12]. SPAdes [13] was used to assemble the genome before Kraken2 [14] was used to identify the sample based on the genomic sequence. The sample was identified as *Shigella flexneri*. Sequencing results consisted of approximately 4.5 million reads, for an average depth of coverage of 147x.

After discharge, oral prednisone was weaned rapidly, and the child has remained well with normal echocardiograms. Workup for immunodeficiency was normal. Confirmatory testing of the stool isolate by the public health laboratory identified *Shigella flexneri*, susceptible to ampicillin. The Public Health investigation did not identify any linked cases.

## Discussion

We report the case of a child with febrile multisystemic illness, including prominent gastrointestinal symptoms, severe hemodynamic compromise, and biochemical inflammation, and SARS-CoV-2 exposure 2 months prior. The clinical picture was consistent with MIS-C, and he was managed accordingly. However, stools were positive for *Shigella flexneri* by molecular and culture-based methods, suggesting a role for this pathogen in his presentation.

We believe that the positive *Shigella* result represented an active infection, rather than asymptomatic carriage. While the child had no obvious exposures to *Shigella* (i.e., no recent travel history nor any link to a documented outbreak), he had typical symptomatology for acute gastroenteritis (fever, vomiting, diarrhea) and positive stool testing by both PCR and culture.

It is possible that the *Shigella* infection entirely accounted for this child’s illness. *Salmonella* and *Campylobacter* infections have been reported to mimic MIS-C, including typhoidal *Salmonella* cases with hemodynamic instability [5], [6], [13]. Gastrointestinal symptoms are common in MIS-C, but stool cultures are insensitive for detecting *Shigella;* sensitivity is doubled by molecular testing but still imperfect [16]. Moreover, infectious stool studies have not been routinely sent in all MIS-C case series [15], [16]. Thus, there may have been missed cases of bacterial gastroenteritis that self-resolved or were treated by brief empiric antibiotic courses.

However, this child’s presentation would be quite atypical for *Shigella* infection. Severe shigellosis is unusual in immunocompetent individuals, and septic shock is exceedingly rare, with few cases reported in the literature [7], [17]. The child did not have any evident risk factors for severe shigellosis, though we cannot formally rule out an occult host immunodeficiency.

Before the *Shigella* result was known, this case was convincing for MIS-C given clinical and laboratory features, COVID-19 exposure 2 months prior, and positive COVID-19 serology without prior immunization. However, some aspects were less typical: there was no mucositis or rash, and the SARS-CoV-2 exposure was 8 weeks prior (rather than 4–6 weeks). The SARS-CoV-2 exposure/infection could have been incidental, in the context of high community rates. Indeed, MIS-C is a very rare complication of COVID-19 infection in children [1]. The rapid clinical response following steroid administration may have been related to non-specific vasopressive effects. At the time, MIS-C management was continued given uncertainty about the *Shigella* PCR result, and the potentially serious consequences of undertreated MIS-C.

It is intriguing to speculate that this child had both *Shigella* gastroenteritis and MIS-C. Similar to KD, some MIS-C case definitions have permitted concurrent infectious diagnoses [17]. Such dual diagnoses may be coincidental, or linked. Dysregulated immune responses have been associated with SARS-CoV-2 infection and MIS-C [1], [18]. In our case, perhaps the recent SARS-CoV-2 infection led to an exaggerated host response to *Shigella*, with a resultant MIS-C phenotype.

This case reinforces the importance of a thorough infectious workup in suspected cases of systemic inflammatory syndromes. Identification of an infectious mimic or a concurrent infection may have implications for treatment or further investigations (e.g., immunodeficiency workup, Public Health investigation). We demonstrate the utility of molecular stool testing and whole genome sequencing to rapidly identify and confirm infectious enterocolitis in critically ill patients. Finally, this case suggests that SARS-CoV-2 may modulate immune responses to subsequent infections.

## CRediT authorship contribution statement

**Donald Brody Duncan:** Writing – review & editing, Data curation, Conceptualization. **Sheridan J.C. Baker:** Writing – review & editing, Writing – original draft, Methodology, Formal analysis, Data curation, Conceptualization. **Rutherford Candy:** Writing – review & editing, Data curation, Conceptualization. **Tania Cellucci:** Writing – review & editing, Supervision, Conceptualization. **Andrew G. McArthur:** Writing – review & editing, Supervision, Methodology, Formal analysis, Data curation, Conceptualization. **Erdman Laura:** Writing – review & editing, Writing – original draft, Conceptualization. **Jeffrey M. Pernica:** Writing – review & editing, Supervision, Conceptualization. **Cheryl Main:** Writing – review & editing, Supervision, Conceptualization. **Marek Smieja:** Writing – review & editing, Supervision, Conceptualization.

## Authors' contributions

All authors conceptualized the manuscript. SJCB performed the whole genome sequencing and data analysis. SJCB and LKE wrote an initial draft of the manuscript. All authors critically revised the manuscript and approved the final version.

## Consent for publication

Written informed consent was obtained from the patient’s legal guardian for publication of this case report. A copy of the written consent is available for review by the Editor-in-Chief of this journal on request.

## Ethics approval and consent to participate

Research Ethics Board approval was not required for this case report.

## Declaration of Competing Interest

The authors declare that they have no known competing financial interests or personal relationships that could have appeared to influence the work reported in this paper.

## Data Availability

All sequencing reads have been deposited in NCBI BioProject accession number PRJNA1225204.
